# MicroRNAs as the cause of schizophrenia in 22q11.2 deletion carriers, and possible implications for idiopathic disease: a mini-review

**DOI:** 10.3389/fnmol.2013.00047

**Published:** 2013-12-05

**Authors:** Andreas J. Forstner, Franziska Degenhardt, Gerhard Schratt, Markus M. Nöthen

**Affiliations:** ^1^Institute of Human Genetics, University of BonnBonn, Germany; ^2^Department of Genomics, Life and Brain CenterBonn, Germany; ^3^Institute of Physiological Chemistry, Philipps-University MarburgMarburg, Germany

**Keywords:** 22q11.2 deletion syndrome, schizophrenia, microRNA, *MIR185*, *DGCR8*, copy number variants, genetic risk factor

## Abstract

The 22q11.2 deletion is the strongest known genetic risk factor for schizophrenia. Research has implicated microRNA-mediated dysregulation in 22q11.2 deletion syndrome (22q11.2DS) schizophrenia-risk. Primary candidate genes are *DGCR8* (DiGeorge syndrome critical region gene 8), which encodes a component of the microprocessor complex essential for microRNA biogenesis, and *MIR185*, which encodes microRNA 185. Mouse models of 22q11.2DS have demonstrated alterations in brain microRNA biogenesis, and that *DGCR8* haploinsufficiency may contribute to these alterations, e.g., via down-regulation of a specific microRNA subset. *miR-185* was the top-scoring down-regulated microRNA in both the prefrontal cortex and the hippocampus, brain areas which are the key foci of schizophrenia research. This reduction in *miR-185* expression contributed to dendritic and spine development deficits in hippocampal neurons. In addition, *miR-185* has two validated targets (RhoA, Cdc42), both of which have been associated with altered expression levels in schizophrenia. These combined data support the involvement of *miR-185* and its down-stream pathways in schizophrenia. This review summarizes evidence implicating microRNA-mediated dysregulation in schizophrenia in both 22q11.2DS-related and idiopathic cases.

## INTRODUCTION

The 22q11.2 deletion syndrome (22q11.2DS), also known as the velocardiofacial/DiGeorge syndrome, is a phenotypically heterogenous disease which is caused by a hemizygous microdeletion on the long arm of chromosome 22 in the region q11.2. The overall prevalence is 1 in 2,000–4,000 live births (Murphy et al., 1999; Botto et al., 2003; Robin and Shprintzen, 2005). The disorder is associated with a high risk for psychiatric disorder.

In particular, 22q11.2DS patients have an estimated 20–25% risk for schizophrenia or related psychotic disorders such as schizoaffective disorder (Murphy et al., 1999; Chow et al., 2006; Bassett and Chow, 2008; Philip and Bassett, 2011). The deletion is therefore the strongest known genetic risk factor for schizophrenia (odds ratio = 20.3; Levinson et al., 2011), and accounts for approximately 1–2% of all schizophrenia cases (Karayiorgou et al., 1995, 2010; Bassett and Chow, 2008; International Schizophrenia Consortium, 2008; Stefansson et al., 2008). Individuals with 22q11.2DS have variable cognitive and behavioral deficits (Karayiorgou et al., 2010) including relative impairments in social judgment, motor skills, verbal learning, and executive functioning (Chow et al., 2006; Philip and Bassett, 2011). In addition, adults with a 22q11.2 microdeletion have a two- to threefold increase in the risk of generalized anxiety disorder compared to the general population (Philip and Bassett, 2011). The major clinical features of 22q11.2DS-related schizophrenia are largely indistinguishable from those of the idiopathic disease (Murphy et al., 1999; Chow et al., 2006; Bassett and Chow, 2008). Identification of schizophrenia-risk gene/s in the 22q11.2DS deletion region may therefore generate insights into the pathophysiology of schizophrenia in general (Earls et al., 2012).

The size of the 22q11.2 deletion varies. The majority of 22q11.2 deletions (around 90%) are 3 Mb in size and span approximately 60 known genes, while the remaining 10% are 1.5 Mb in size and encompass around 35 genes (Edelmann et al., 1999; Shaikh et al., 2000). Both the larger and the smaller 22q11.2 microdeletions usually result from non-allelic homologous recombination, which is mediated by flanking low-copy repeats (Edelmann et al., 1999). Although the 22q11.2DS phenotype is highly variable, its severity is not correlated with the size of the deletion. This suggests that the minimal 1.5 Mb deletion region is crucial in terms of etiology (Carlson et al., 1997; Karayiorgou et al., 2010).

Initial research to identify schizophrenia-risk genes in the 22q11.2 deletion region proved unsuccessful. The identification of heterozygous loss-of-function mutations in non-deleted schizophrenia patients would be the most obvious human genetic evidence for the involvement of a specific gene in disease susceptibility. Hopes were raised by the identification of heterozygous point mutations in the T-box 1 gene (*TBX1*), which encodes a T-box transcription factor, that resulted in the characteristic abnormal facies and cardiac defects of 22q11.2DS in patients without a 22q11.2 deletion (Yagi et al., 2003; Zweier et al., 2007). However, no such mutation has yet been identified in schizophrenia patients.

In contrast, recent research in the *Df(16)A*^+/-^ mouse model has generated breakthroughs in our understanding of the underlying biological mechanisms of 22q11.2DS schizophrenia-risk. This engineered mouse strain carries a heterozygous chromosomal deletion which spans a segment syntenic to the human 22q11.2 locus. *Df(16)A*^+/-^ mice show deficits in the synaptic connectivity of hippocampal neurons, including a lower density of dendritic spines and glutamatergic synapses (Mukai et al., 2008). In addition, *Df(16)A*^+/-^ mice display hyperactive behavior and deficits in spatial working memory-dependent learning (Stark et al., 2008). Further characterization of this animal model has suggested that the 22q11.2 microdeletion results in alterations in the biogenesis of brain microRNAs (Stark et al., 2008; Xu et al., 2010). Primary candidate genes in the region are the DiGeorge syndrome critical region gene 8 (*DGCR8*), which encodes a component of the microprocessor complex essential for microRNA biogenesis (Tomari and Zamore, 2005), and the *MIR185* gene (Karayiorgou et al., 2010), which encodes microRNA 185. Both genes are located within the minimal 1.5 Mb deletion region at 22q11.2 (Karayiorgou et al., 2010).

The microRNAs are a class of 21–25-nucleotide small non-coding RNAs. They control the expression of their target genes by binding to target sites in messenger RNAs (mRNAs), typically in their 3′ untranslated regions (He and Hannon, 2004; Meola et al., 2009). In most cases, microRNAs negatively regulate target gene expression through a combination of repression of mRNA translation and promotion of mRNA decay. Each microRNA usually controls up to several hundred target mRNAs, while one mRNA target can be synergistically regulated by multiple microRNAs (Sathyan et al., 2007; Didiano and Hobert, 2008; Drew et al., 2011). This allows microRNAs to integrate different intracellular signals and to regulate various signaling pathways (Johnston and Hobert, 2003; Choi et al., 2007). Accumulating evidence suggests that microRNAs contribute to the basic mechanisms underlying brain development and plasticity (**Table 1**; Fineberg et al., 2009; Schratt, 2009; Im and Kenny, 2012). Neural microRNAs play an important role at various stages of synaptic development, including dendritic arborization (Vo et al., 2005; Yu et al., 2008), synapse formation, and synapse maturation (Caygill and Johnston, 2008; Siegel et al., 2009). Arguably the two most extensively studied examples in the context of synapse development are *miR-132* and *miR-134*. CREB-induced *miR-132* promotes dendritogenesis and spine growth by down-regulating p250GAP (Wayman et al., 2008; Magill et al., 2010). *miR-134* on the other hand is required for activity-dependent dendritic arborization and the restriction of spine growth by targeting Pumilio-2 and Lim-domain containing protein kinase (Limk1), respectively (Schratt et al., 2006; Fiore et al., 2009). Furthermore, investigation of a mouse model displaying conditional knock-out of the microRNA biogenesis enzyme *Dicer* (Schratt, 2009) revealed disrupted morphogenesis of the hippocampus and cortex (Davis et al., 2008), suggesting that undisturbed microRNA processing might be necessary for normal brain development (Xu et al., 2010). These data suggest the possible involvement of microRNA-dependent dysregulation in the pathogenesis of various psychiatric disorders (Forero et al., 2010; Xu et al., 2010), including schizophrenia (Beveridge et al., 2008).

**Table 1:** List of individual microRNAs involved in neural development and synapse development/plasticity and their mRNA targets in mice and men.

MicroRNA	Function	Target/s	Reference
**microRNAs involved in neural development**
let-7	Promotes neuronal differentiation	HMGA, LIN28, TLX	Nishino et al. (2008), Rybak et al. (2008), Zhao et al. (2010)
	Neural tube closure	MLIN41	Maller Schulman et al. (2008)
miR-7a	Inhibits differentiation of forebrain dopaminergic neurons	PAX6	de Chevigny et al. (2012)
miR-9	Promotes neuronal differentiation	FOXG1, TLX, STAT3, REST, FGF8, FGFR1, FOXP2	Krichevsky et al. (2006), Leucht et al. (2008), Packer et al. (2008), Shibata et al. (2008),^2011^, Zhao et al. (2009), Yoo et al. (2011), Clovis et al. (2012)
	Promotes proliferation of early human embryonic stem cell-derived neural progenitor cells	STMN1	Delaloy et al. (2010)
miR-9*	Promotes neuronal differentiation	BAF53a	Yoo et al. (2009),^2011^
	?	coREST	Packer et al. (2008)
miR-17	Inhibits neural differentiation	?	Beveridge et al. (2009)
miR-17/92	Promotes axonal outgrowth in embryonic cortical neurons	PTEN	Zhang et al. (2013)
	Controls spinal neural progenitor patterning	Olig2	Chen et al. (2011b)
miR-34a	Antagonizes neuronal differentiation	Numbl	Fineberg et al. (2012)
	Promotes neuroblastoma and medulloblastoma differentiation	?	Agostini et al. (2011), de Antonellis et al. (2011)
miR-92b	Limits the production of intermediate cortical progenitors	?	Nowakowski et al. (2013)
miR-124	Promotes neuronal differentiation	SCP1, PTBP1, SOX9, DLX2, JAG1, BAF53a, RhoG, Lhx2	Makeyev et al. (2007), Visvanathan et al. (2007), Cheng et al. (2009), Yoo et al. (2009),^2011^, Sanuki et al. (2011), Akerblom et al. (2012), Franke et al. (2012)
miR-125	Promotes neuronal differentiation	GLI1, SMO, LIN28, SMAD4	Ferretti et al. (2008), Rybak et al. (2008), Boissart et al. (2012)
miR-128	Inhibits NSC proliferation	BMI1	Godlewski et al. (2008)
miR-132	Promotes synaptic integration and survival of newborn dentate gyrus and olfactory bulb neurons	Nurr1, FoxP2	Luikart et al. (2011), Clovis et al. (2012), Pathania et al. (2012)
	Promotes differentiation of dopamine neurons	Nurr1	Yang et al. (2012)
miR-133b	Modulates maturation of dopaminergic neurons	PITX3	Kim et al. (2007)
miR-137	Promotes neural differentiation of embryonic stem cells	Klf4, Tbx3	Jiang et al. (2013)
miR-200	Promotes olfactory progenitor differentiation	SOX2, ETF3	Choi et al. (2008), Peng et al. (2012)
miR-324-5p	Promotes neuronal differentiation	GLI1, SMO	Ferretti et al. (2008)
miR-326	Promotes neuronal differentiation	GLI1, SMO	Ferretti et al. (2008)
miR-541	Promotes neurite outgrowth of PC12 cells	Synapsin-1	Zhang et al. (2011)
**microRNAs involved in synapse development/plasticity**
miR-29a/b	Inhibits spine maturation	Arpc3	Lippi et al. (2011)
miR-34c	Negative constraint of memory consolidation	SIRT1	Zovoilis et al. (2011)
miR-124	Regulates neuronal process complexity	RhoG, Cdc42	Yu et al. (2008), Franke et al. (2012)
miR-125a	Reduces number of branched spines	PSD-95	Muddashetty et al. (2011)
miR-125b	Negatively regulates spine morphology	NR2A	Edbauer et al. (2010)
miR-129	Reduces intrinsic neuronal excitability	Kv1.1	Sosanya et al. (2013)
miR-132	Promotes dendritogenesis Promotes spine growth Facilitates memory acquisition Positively regulates LTP Essential for experience-dependent plasticity in visual cortex Negatively regulates circadian clock resetting	P250RhoGap, MeCP2, RFX4	Vo et al. (2005), Cheng et al. (2007), Wayman et al. (2008), Edbauer et al. (2010), Impey et al. (2010), Mellios et al. (2011), Tognini et al. (2011), Scott et al. (2012), Hansen et al. (2013), Remenyi et al. (2013), Wang et al. (2013)
miR-134	Necessary for activity-dependent dendritogenesis	Pum2	Fiore et al. (2009)
	Restricts spine growth	Limk1	Schratt et al. (2006)
	Interferes with memory formation and LTP	Creb1	Gao et al. (2010)
miR-137	Inhibits dendritic morphogenesis	Mib1	Smrt et al. (2010)
miR-138	Negatively regulates dendritic spine size	Apt-1	Siegel et al. (2009)
	Represses axon regeneration	SIRT1	Liu et al. (2013)
miR-146a	Inhibits AMPAR endocytosis	MAP1B	Chen and Shen (2013)
miR-181a	Reduces AMPAR expression and spine formation	GluA2	Saba et al. (2012)
miR-188	Controls dendritic plasticity	Nrp-2	Lee et al. (2012)
miR-219	Regulates circadian clock length	SCOP	Cheng et al. (2007)
miR-375	Reduces dendrite density	HuD	Abdelmohsen et al. (2010)
miR-483-5p	Rescues dendritic spine defects in MeCP2-overexpressing neurons	MeCP2	Han et al. (2013)
miR-485	Regulates presynaptic homeostatic plasticity	Synapsin-1	Cohen et al. (2011)

*NSC, neural stem cell; LTP, long-term potentiation; ? – unknown.*

The present review summarizes the various lines of evidence implicating microRNAs as the causal factor for schizophrenia in 22q11.2DS carriers and emerging evidence from expression studies and genome-wide association studies (GWAS) that these mechanisms may also be involved in the development of idiopathic schizophrenia.

## THE ROLE OF *DGCR8*

Investigation of *Dgcr8*^+/-^ mice confirmed that heterozygous *Dgcr8* deficiency was responsible for the reduced biogenesis of microRNAs observed in *Df(16)A*^+/-^ mice (Stark et al., 2008; Schofield et al., 2011). *Dgcr8*^+/-^ mice displayed 22q11.2DS-associated cognitive and behavioral deficits, and altered short-term plasticity in the prefrontal cortex (PFC; Stark et al., 2008). This indicates that *DGCR8* heterozygosity, and the resulting alterations in microRNA expression, are sufficient to produce some of the neural deficits observed in 22q11.2DS (Schofield et al., 2011). On the neuronal cell level, *Dgcr8* deficiency resulted in structural changes in dendritic spines and reduced dendritic complexity in the hippocampus (Stark et al., 2008). Schofield et al. (2011) identified alterations in the electrical properties of layer V pyramidal neurons in the medial PFC of *Dgcr8*^+/-^ mice, as well as a decrease in the complexity of the basal dendrites and reduced excitatory synaptic transmission. These functional results suggest that precise microRNA expression is critical for the development of PFC circuitry (Schofield et al., 2011), circuitry which has been reported to be altered in schizophrenia patients (Ursu et al., 2011).

*Dgcr8*^+/-^ mice also displayed a decrease in the number of cortical neurons, structural deficits in dendritic spines in the PFC, and alterations in synaptic potentiation and short-term plasticity (Fenelon et al., 2011). These alterations might influence functional connectivity (Schreiner et al., 2013), and could be implicated in the observed cognitive and behavioral deficits. In particular, they may explain observed alterations in prepulse inhibition (Stark et al., 2008), which have also been reported in schizophrenia patients (Powell et al., 2009).

Ouchi et al. (2013) showed that heterozygous *Dgcr8* deficiency in mice led to reduced progenitor cell proliferation and neurogenesis in the adult hippocampus. This is of particular interest since alterations in the anatomy, histology, and function of the hippocampus have been consistently reported in schizophrenia patients (Tamminga et al., 2010). Several schizophrenia-associated genes were down-regulated in the hippocampus of *Dgcr8*^+/-^ mice (Ouchi et al., 2013), including the insulin-like growth factor 2 (IGF2), which was recently found to play a crucial role in hippocampal functions such as memory consolidation and fear extinction (Agis-Balboa et al., 2011; Chen et al., 2011a). Interestingly, restoration of IGF2 expression in the hippocampus rescued the observed spatial working memory deficits in *Dgcr8*^+/-^ mice, suggesting that IGF2 contributes – at least in part – to the learning and spatial working memory deficits that are associated with 22q11.2DS-related schizophrenia (Ouchi et al., 2013).

The question now arises as to which specific microRNAs are regulated by *DGCR8*. The investigation of *Dgcr8*^+/-^ mice identified 59 down-regulated microRNAs in the PFC and 30 down-regulated microRNAs in the hippocampus (Stark et al., 2008). These down-regulated microRNAs include *miR-185*, which is also located in the minimal 1.5 Mb deletion region at 22q11.2.

## THE ROLE OF *MIR185*

**FIGURE 1:**
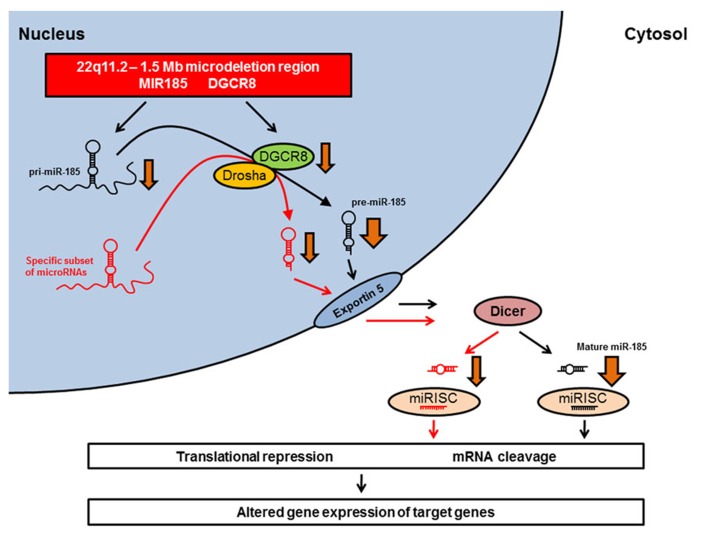
**Dysregulation of microRNA biogenesis in 22q11.2DS animal models.** The *MIR185* and the *DGCR8* genes are located within the minimal 1.5 Mb microdeletion region on chromosome 22q11.2 and the equivalent region of mouse chromosome 16. The microdeletion leads to a hemizygosity of *MIR185* and *DGCR8*. The heterozygous *Dgcr8* deficiency is responsible for the reduced biogenesis of a specific subset of microRNAs (red) observed in *Df(16)A*^+/-^ mice (Stark et al., 2008). These down-regulated microRNAs include *miR-185* (black). The pronounced reduction of *miR-185* expression (indicated by a broader arrow) may be due to a combined effect of the hemizygosity of the *MIR185* gene and the impaired maturation of the *pri-miR-185* transcript secondary to reduced *Dgcr8* levels (Xu et al., 2013). The resulting alterations in mature microRNA expression levels may lead to altered gene expression of target genes, which might produce some of the neural, cognitive, and behavioral deficits observed in 22q11.2DS. miRISC, microRNA-induced silencing complex.

Studies of 22q11.2DS mouse models have identified *miR-185* as the top-scoring down-regulated microRNA in schizophrenia-associated brain areas (Stark et al., 2008; Benetti et al., 2009). A recent study by Xu et al. (2013) confirmed the drastic reduction in *miR-185* expression levels in the hippocampus and PFC of *Df(16)A*^+/-^ mice, and showed that this reduction contributed to deficits in dendritic complexity and spine development in hippocampal neurons. In addition, *Dgcr8* deficiency resulted in an approximately 20% reduction in *miR-185* expression in the hippocampus (Xu et al., 2013). This suggests that the pronounced reduction of *miR-185* expression in *Df(16)A*^+/-^ mice – a reduction which is much more pronounced than would be expected by the 50% decrease in gene dosage – may be due to the combined effect of the hemizygosity of the *MIR185* gene and the impaired maturation of the *pri-miR-185* transcript secondary to reduced *Dgcr8* levels (**Figure 1**; Xu et al., 2013). The large reduction in *miR-185* expression renders *miR-185* unique among the genes that are affected by the 22q11.2 microdeletion (Xu et al., 2013).

A recent human study confirmed a down-regulation of *MIR185* expression to 0.4× normal levels in the peripheral blood of patients with 22q11.2DS (de la Morena et al., 2013). This finding suggests that pronounced *miR-185* down-regulation also occurs in patients with 22q11.2DS.

Previous research has shown that *MIR185* is present or enriched in synapses (Lugli et al., 2008; Earls et al., 2012). This may indicate that *MIR185* is of relevance to neural function, since a number of microRNAs have been shown to play a critical role in synaptic plasticity (Schratt, 2009).

Earls et al. (2012) identified *MIR185* as a regulator of sarco(endo)plasmic reticulum Ca(2+) ATPase (SERCA2) which maintains Ca^2^^+^ levels in the endoplasmatic reticulum. The depletion of *MIR185* contributes to SERCA2 upregulation and has been proposed as a mechanism leading to abnormal hippocampal synaptic plasticity in 22q11.2DS mouse models. The microRNA regulation of SERCA2 translation may also be implicated in the elevation of SERCA2 protein observed in the post-mortem brains of idiopathic schizophrenia patients (Earls et al., 2012). These results suggest that microRNA-mediated SERCA2 upregulation at central synapses might be a mechanistic link between 22q11.2DS and idiopathic schizophrenia (Earls et al., 2012).

Further support for the involvement of *MIR185* in schizophrenia is provided by findings that two of its validated targets (RhoA, Cdc42; Liu et al., 2011) are associated with altered expression levels in schizophrenia (Hill et al., 2006; Ide and Lewis, 2010). Cdc42 (cell division cycle 42) is a member of the RhoGTPase family (Hill et al., 2006) and promotes dendritic spine formation (Irie and Yamaguchi, 2002; Tada and Sheng, 2006; Wegner et al., 2008) by regulating the polymerization of the actin cytoskeleton into filopodia (Nobes and Hall, 1995). Cdc42 is activated by Collybistin/ARHGEF9 (Reid et al., 1999; Reddy-Alla et al., 2010), which has recently been identified as a candidate blood biomarker in psychosis (Kurian et al., 2011). RhoA (Ras homologous member A) also belongs to the RhoGTPase family and regulates the destabilization of the actin cytoskeleton (Hill et al., 2006). The activation of RhoA leads to a reduction in the number of dendritic branches and the density of dendritic spines (Nakayama et al., 2000; Hill et al., 2006).

## EXPRESSION OF microRNAs IN IDIOPATHIC SCHIZOPHRENIA

Post-mortem studies of human brain tissue have revealed alterations in microRNA expression in patients with schizophrenia. This research is reviewed elsewhere (Beveridge and Cairns, 2012). Briefly, numerous microRNAs have been implicated in the disorder across multiple studies, including 16 microRNAs with increased and 11 microRNAs with decreased expression (Beveridge and Cairns, 2012). Of particular interest in the context of the present review is the study by Moreau et al. (2011). This reported a significant overlap between microRNAs dysregulated in human post-mortem brain tissue and microRNAs previously found to be altered in the PFC of a 22q11.2DS mouse model (Stark et al., 2008; Moreau et al., 2011). This finding supports the hypothesis that findings in 22q11.2DS might be of relevance to idiopathic schizophrenia (Brzustowicz and Bassett, 2012).

## GWAS OF IDIOPATHIC SCHIZOPHRENIA

The involvement of microRNA-dependent dysregulation in schizophrenia is supported by the results of the large GWAS of schizophrenia performed by the Schizophrenia Psychiatric Genome-Wide Association Study (GWAS) Consortium (2011). In total 17,836 patients and 33,859 controls were investigated. A single-nucleotide polymorphism (SNP) in an intron of *MIR137* was the second strongest finding (odds ratio = 1.12). Four other loci with genome-wide significance in this study contained predicted targets of *MIR137* (*TCF4*, *CACNA1C*, *CSMD1*, *C10orf26*). All four genes have recently been validated as *miR-137* targets (Kwon et al., 2013).

The miRanda database lists 5,487 genes as targets of *miR-137* (John et al., 2004). Interestingly, *ZNF804A* is listed as a validated target (Kim et al., 2012a). This gene has shown strong association with schizophrenia in previous studies (O’Donovan et al., 2008; Williams et al., 2011). Other promising targets include the ubiquitin ligase Mind bomb one (Mib1; Smrt et al., 2010) which plays an important role in neurogenesis and neurodevelopment (Itoh et al., 2003; Choe et al., 2007; Ossipova et al., 2009).

Research in post-mortem brain samples suggests that the functional effect of the *miR-137* risk allele may result in a reduced *miR-137* expression (Guella et al., 2013). Further down-stream this may be responsible for the reduced white matter integrity, smaller hippocampi, and larger lateral ventricles observed in schizophrenia patients with the *miR-137* risk genotype (Lett et al., 2013).

## CONCLUSION AND OUTLOOK

Strong evidence suggests that microRNA dysregulation is implicated in the development of schizophrenia in 22q11.2DS patients. This is consistent with the growing recognition of microRNAs as important regulators of gene expression. As microRNAs integrate different intracellular signals and regulate various signaling pathways (Johnston and Hobert, 2003; Choi et al., 2007), the dysregulation of specific microRNAs could lead to the heterogenous phenotype observed in 22q11.2DS.

As summarized above, emerging evidence from expression and genetic analyses suggests that the same microRNA-regulated pathways may also play a role in idiopathic schizophrenia. However, despite a number of systematic investigations of genes in the 22q11.2DS region and the ever increasing number of GWAS data sets (Karayiorgou et al., 2010; Sullivan et al., 2012), no genetic study to date has identified common variation in *DGCR8* or *MIR185* as a risk factor for schizophrenia. This may simply reflect a lack of common functional variants at these loci. This hypothesis is supported by a recent study of genetic regulation of microRNA expression (Gamazon et al., 2012). Gamazon et al. (2012) systematically investigated the relationship between microRNA expression levels (as quantitative traits) and common genetic variation. In this study, no SNP had significant *cis* effects on *miR-185* expression. A small number of SNPs have been reported to have significant *cis* effects on *DGCR8* expression in human monocytes (Zeller et al., 2010), and fibroblasts (Dimas et al., 2009). However, these associations might be tissue-specific, since a recent study of five different human post-mortem brain regions failed to identify any SNP with significant cis effects on *DGCR8* expression (Kim et al., 2012b).

A challenge for future research will be to identify and validate the target genes that are affected by microRNA dysregulation and their respective pathways in a more comprehensive manner (Drew et al., 2011). Such research will improve our understanding of how alterations in microRNA-regulated genetic networks contribute to the pathophysiology of both 22q11.2DS-related and idiopathic schizophrenia.

Idiopathic schizophrenia is a multifactorial disorder for which both genetic and environmental factors exert an impact on disease susceptibility (Sawa and Snyder, 2002). However, very few data are available concerning the influence of environmental factors on microRNA dysregulation. Recent studies in mice showed that environmental factors such as stress resulted in alterations of microRNA expression in the frontal cortex (Rinaldi et al., 2010). Future studies are therefore warranted to investigate the extent to which environmental factors are associated with microRNA dysregulation in schizophrenia.

Further research into the precise role of microRNAs in schizophrenia is important clinically, since modification of microRNA dysregulation would represent a novel therapeutic approach to this devastating and chronic disease. MicroRNAs are excellent candidates for therapy since they regulate multiple targets in various signaling pathways, thereby minimizing the risk of resistance development or compensatory mechanisms (Soriano et al., 2013). This view is supported by several recent studies and reviews, which have highlighted microRNAs as promising pharmacological targets in the treatment of complex diseases such as psychiatric disorders (Im and Kenny, 2012), cancer (Soriano et al., 2013), and diabetes (Mao et al., 2013).

## Conflict of Interest Statement

The authors declare that the research was conducted in the absence of any commercial or financial relationships that could be construed as a potential conflict of interest.

## References

[B1] Abdelmohsen K. Hutchison E. R. Lee E. K. Kuwano Y. Kim M. M. Masuda K. (2010). miR-375 inhibits differentiation of neurites by lowering HuD levels. *Mol. Cell. Biol.* 30 4197–4210 10.1128/MCB.00316-10 20584986 PMC2937556

[B2] Agis-Balboa R. C. Arcos-Diaz D. Wittnam J. Govindarajan N. Blom K. Burkhardt S. (2011). A hippocampal insulin-growth factor 2 pathway regulates the extinction of fear memories. *EMBO J.* 30 4071–4083 10.1038/emboj.2011.293 21873981 PMC3209781

[B3] Agostini M. Tucci P. Killick R. Candi E. Sayan B. S. Rivetti Di Val Cervo P. (2011). Neuronal differentiation by TAp73 is mediated by microRNA-34a regulation of synaptic protein targets. *Proc. Natl. Acad. Sci. U.S.A.* 108 21093–21098 10.1073/pnas.1112061109 22160687 PMC3248477

[B4] Akerblom M. Sachdeva R. Jakobsson J. (2012). Functional studies of microRNAs in neural stem cells: problems and perspectives. *Front. Neurosci.* 6 14 10.3389/fnins.2012.00014 PMC3273711 22347160

[B5] Bassett A. S. Chow E. W. (2008). Schizophrenia and 22q11.2 deletion syndrome. *Curr. Psychiatry Rep.* 10 148–157 10.1007/s11920-008-0026-1 18474208 PMC3129332

[B6] Benetti S. Mechelli A. Picchioni M. Broome M. Williams S. Mcguire P. (2009). Functional integration between the posterior hippocampus and prefrontal cortex is impaired in both first episode schizophrenia and the at risk mental state. *Brain* 132 2426–2436 10.1093/brain/awp098 19420091

[B7] Beveridge N. J. Cairns M. J. (2012). MicroRNA dysregulation in schizophrenia. *Neurobiol. Dis.* 46 263–271 10.1016/j.nbd.2011.12.029 22207190

[B8] Beveridge N. J. Tooney P. A. Carroll A. P. Gardiner E. Bowden N. Scott R. J. (2008). Dysregulation of miRNA 181b in the temporal cortex in schizophrenia. *Hum. Mol. Genet.* 17 1156–1168 10.1093/hmg/ddn005 18184693

[B9] Beveridge N. J. Tooney P. A. Carroll A. P. Tran N. Cairns M. J. (2009). Down-regulation of miR-17 family expression in response to retinoic acid induced neuronal differentiation. *Cell. Signal.* 21 1837–1845 10.1016/j.cellsig.2009.07.019 19666108

[B10] Boissart C. Nissan X. Giraud-Triboult K. Peschanski M. Benchoua A. (2012). miR-125 potentiates early neural specification of human embryonic stem cells. *Development* 139 1247–1257 10.1242/dev.073627 22357933

[B11] Botto L. D. May K. Fernhoff P. M. Correa A. Coleman K. Rasmussen S. A. (2003). A population-based study of the 22q11.2 deletion: phenotype, incidence, and contribution to major birth defects in the population. *Pediatrics* 112 101–107 10.1542/peds.112.1.101 12837874

[B12] Brzustowicz L. M. Bassett A. S. (2012). miRNA-mediated risk for schizophrenia in 22q11.2 deletion syndrome. * Front. Genet.* 3 291 10.3389/fgene.2012.00291 PMC3521194 23248646

[B13] Carlson C. Sirotkin H. Pandita R. Goldberg R. Mckie J. Wadey R. (1997). Molecular definition of 22q11 deletions in 151 velo-cardio-facial syndrome patients. *Am. J. Hum. Genet.* 61 620–629 10.1086/515508 9326327 PMC1715959

[B14] Caygill E. E. Johnston L. A. (2008). Temporal regulation of metamorphic processes in *Drosophila* by the let-7 and miR-125 heterochronic microRNAs. *Curr. Biol.* 18 943–950 10.1016/j.cub.2008.06.020 18571409 PMC2736146

[B15] Chen D. Y. Stern S. A. Garcia-Osta A. Saunier-Rebori B. Pollonini G. Bambah-Mukku D. (2011a). A critical role for IGF-II in memory consolidation and enhancement. *Nature* 469 491–497 10.1038/nature09667 21270887 PMC3908455

[B16] Chen J. A. Huang Y. P. Mazzoni E. O. Tan G. C. Zavadil J. Wichterle H. (2011b). Mir-17-3p controls spinal neural progenitor patterning by regulating Olig2/Irx3 cross-repressive loop. *Neuron* 69 721–735 10.1016/j.neuron.2011.01.014 21338882 PMC3062262

[B17] Chen Y. L. Shen C. K. (2013). Modulation of mGluR-dependent MAP1B translation and AMPA receptor endocytosis by microRNA miR-146a-5p. *J. Neurosci.* 33 9013–9020 10.1523/JNEUROSCI.5210-12.2013 23699512 PMC6705019

[B18] Cheng H. Y. Papp J. W. Varlamova O. Dziema H. Russell B. Curfman J. P. (2007). microRNA modulation of circadian-clock period and entrainment. *Neuron* 54 813–829 10.1016/j.neuron.2007.05.017 17553428 PMC2590749

[B19] Cheng L. C. Pastrana E. Tavazoie M. Doetsch F. (2009). miR-124 regulates adult neurogenesis in the subventricular zone stem cell niche. *Nat. Neurosci.* 12 399–408 10.1038/nn.2294 19287386 PMC2766245

[B20] Choe E. A. Liao L. Zhou J. Y. Cheng D. Duong D. M. Jin P. (2007). Neuronal morphogenesis is regulated by the interplay between cyclin-dependent kinase 5 and the ubiquitin ligase mind bomb 1. *J. Neurosci.* 27 9503–9512 10.1523/JNEUROSCI.1408-07.2007 17728463 PMC6673137

[B21] Choi P. S. Zakhary L. Choi W. Y. Caron S. Alvarez-Saavedra E. Miska E. A. (2008). Members of the miRNA-200 family regulate olfactory neurogenesis. *Neuron* 57 41–55 10.1016/j.neuron.2007.11.018 18184563 PMC2204047

[B22] Choi W. Y. Giraldez A. J. Schier A. F. (2007). Target protectors reveal dampening and balancing of Nodal agonist and antagonist by miR-430. *Science* 318 271–274 10.1126/science.1147535 17761850

[B23] Chow E. W. Watson M. Young D. A. Bassett A. S. (2006). Neurocognitive profile in 22q11 deletion syndrome and schizophrenia. *Schizophr. Res.* 87 270–278 10.1016/j.schres.2006.04.007 16753283 PMC3127863

[B24] Clovis Y. M. Enard W. Marinaro F. Huttner W. B De Pietri Tonelli D. (2012). Convergent repression of Foxp2 3′UTR by miR-9 and miR-132 in embryonic mouse neocortex: implications for radial migration of neurons. *Development* 139 3332–3342 10.1242/dev.078063 22874921

[B25] Cohen J. E. Lee P. R. Chen S. Li W. Fields R. D. (2011). MicroRNA regulation of homeostatic synaptic plasticity. *Proc. Natl. Acad. Sci. U.S.A.* 108 11650–11655 10.1073/pnas.1017576108 21697510 PMC3136313

[B26] Davis T. H. Cuellar T. L. Koch S. M. Barker A. J. Harfe B. D. Mcmanus M. T. (2008). Conditional loss of Dicer disrupts cellular and tissue morphogenesis in the cortex and hippocampus. *J. Neurosci.* 28 4322–4330 10.1523/JNEUROSCI.4815-07.2008 18434510 PMC3844796

[B27] de Antonellis P. Medaglia C. Cusanelli E. Andolfo I. Liguori L. De Vita G. (2011). MiR-34a targeting of Notch ligand delta-like 1 impairs CD15^+^/CD133^+^ tumor-propagating cells and supports neural differentiation in medulloblastoma. *PLoS ONE* 6: e24584 10.1371/journal.pone.0024584 PMC3171461 21931765

[B28] de Chevigny A. Core N. Follert P. Gaudin M. Barbry P. Beclin C. (2012). miR-7a regulation of Pax6 controls spatial origin of forebrain dopaminergic neurons. *Nat. Neurosci.* 15 1120–1126 10.1038/nn.3142 22729175

[B29] Delaloy C. Liu L. Lee J. A. Su H. Shen F. Yang G. Y. (2010). MicroRNA-9 coordinates proliferation and migration of human embryonic stem cell-derived neural progenitors. *Cell Stem Cell* 6 323–335 10.1016/j.stem.2010.02.015 20362537 PMC2851637

[B30] de la Morena M. T. Eitson J. L. Dozmorov I. M. Belkaya S. Hoover A. R. Anguiano E. (2013). Signature MicroRNA expression patterns identified in humans with 22q11.2 deletion/DiGeorge syndrome. * Clin. Immunol.* 147 11–22 10.1016/j.clim.2013.01.011 23454892 PMC3748608

[B31] Didiano D. Hobert O. (2008). Molecular architecture of a miRNA-regulated 3′ UTR. *RNA* 14 1297–1317 10.1261/rna.1082708 18463285 PMC2441980

[B32] Dimas A. S. Deutsch S. Stranger B. E. Montgomery S. B. Borel C. Attar-Cohen H. (2009). Common regulatory variation impacts gene expression in a cell type-dependent manner. *Science* 325 1246–1250 10.1126/science.1174148 19644074 PMC2867218

[B33] Drew L. J. Crabtree G. W. Markx S. Stark K. L. Chaverneff F. Xu B. (2011). The 22q11.2 microdeletion: fifteen years of insights into the genetic and neural complexity of psychiatric disorders. * Int. J. Dev. Neurosci.* 29 259–281 10.1016/j.ijdevneu.2010.09.007 20920576 PMC3074020

[B34] Earls L. R. Fricke R. G. Yu J. Berry R. B. Baldwin L. T. Zakharenko S. S. (2012). Age-dependent microRNA control of synaptic plasticity in 22q11 deletion syndrome and schizophrenia. *J. Neurosci.* 32 14132–14144 10.1523/JNEUROSCI.1312-12.2012 23055483 PMC3486522

[B35] Edbauer D. Neilson J. R. Foster K. A. Wang C. F. Seeburg D. P. Batterton M. N. (2010). Regulation of synaptic structure and function by FMRP-associated microRNAs miR-125b and miR-132. *Neuron* 65 373–384 10.1016/j.neuron.2010.01.005 20159450 PMC5018398

[B36] Edelmann L. Pandita R. K. Morrow B. E. (1999). Low-copy repeats mediate the common 3-Mb deletion in patients with velo-cardio-facial syndrome. *Am. J. Hum. Genet.* 64 1076–1086 10.1086/302343 10090893 PMC1377832

[B37] Fenelon K. Mukai J. Xu B. Hsu P. K. Drew L. J. Karayiorgou M. (2011). Deficiency of Dgcr8, a gene disrupted by the 22q11.2 microdeletion, results in altered short-term plasticity in the prefrontal cortex. * Proc. Natl. Acad. Sci. U.S.A.* 108 4447–4452 10.1073/pnas.1101219108 21368174 PMC3060227

[B38] Ferretti E. De Smaele E. Miele E. Laneve P. Po A. Pelloni M. (2008). Concerted microRNA control of Hedgehog signalling in cerebellar neuronal progenitor and tumour cells. *EMBO J.* 27 2616–2627 10.1038/emboj.2008.172 18756266 PMC2567402

[B39] Fineberg S. K. Datta P. Stein C. S. Davidson B. L. (2012). MiR-34a represses Numbl in murine neural progenitor cells and antagonizes neuronal differentiation. *PLoS ONE* 7:e38562 10.1371/journal.pone.0038562 PMC3372529 22701667

[B40] Fineberg S. K. Kosik K. S. Davidson B. L. (2009). MicroRNAs potentiate neural development. *Neuron* 64 303–309 10.1016/j.neuron.2009.10.020 19914179

[B41] Fiore R. Khudayberdiev S. Christensen M. Siegel G. Flavell S. W. Kim T. K. (2009). Mef2-mediated transcription of the miR379-410 cluster regulates activity-dependent dendritogenesis by fine-tuning Pumilio2 protein levels. *EMBO J.* 28 697–710 10.1038/emboj.2009.10 19197241 PMC2647767

[B42] Forero D. A. Van Der Ven K. Callaerts P. Del-Favero J. (2010). miRNA genes and the brain: implications for psychiatric disorders. *Hum. Mutat.* 31 1195–1204 10.1002/humu.21344 20725930

[B43] Franke K. Otto W. Johannes S. Baumgart J. Nitsch R. Schumacher S. (2012). miR-124-regulated RhoG reduces neuronal process complexity via ELMO/Dock180/Rac1 and Cdc42 signalling. *EMBO J.* 31 2908–2921 10.1038/emboj.2012.130 22588079 PMC3395090

[B44] Gamazon E. R. Ziliak D. Im H. K. Lacroix B. Park D. S. Cox N. J. (2012). Genetic architecture of microRNA expression: implications for the transcriptome and complex traits. *Am. J. Hum. Genet.* 90 1046–1063 10.1016/j.ajhg.2012.04.023 22658545 PMC3370272

[B45] Gao J. Wang W. Y. Mao Y. W. Graff J. Guan J. S. Pan L. (2010). A novel pathway regulates memory and plasticity via SIRT1 and miR-134. *Nature* 466 1105–1109 10.1038/nature09271 20622856 PMC2928875

[B46] Godlewski J. Nowicki M. O. Bronisz A. Williams S. Otsuki A. Nuovo G. (2008). Targeting of the Bmi-1 oncogene/stem cell renewal factor by microRNA-128 inhibits glioma proliferation and self-renewal. *Cancer Res.* 68 9125–9130 10.1158/0008-5472.CAN-08-2629 19010882

[B47] Guella I. Sequeira A. Rollins B. Morgan L. Torri F. Van Erp T. G. (2013). Analysis of miR-137 expression and rs1625579 in dorsolateral prefrontal cortex. *J. Psychiatry Res.* 47 1215–1221 10.1016/j.jpsychires.2013.05.021 PMC3753093 23786914

[B48] Han K. Gennarino V. A. Lee Y. Pang K. Hashimoto-Torii K. Choufani S. (2013). Human-specific regulation of MeCP2 levels in fetal brains by microRNA miR-483-5p. *Genes Dev.* 27 485–490 10.1101/gad.207456.112 23431031 PMC3605462

[B49] Hansen K. F. Karelina K. Sakamoto K. Wayman G. A. Impey S. Obrietan K. (2013). miRNA-132: a dynamic regulator of cognitive capacity. *Brain Struct. Funct.* 218 817–831 10.1007/s00429-012-0431-4 22706759 PMC3508255

[B50] He L. Hannon G. J. (2004). MicroRNAs: small RNAs with a big role in gene regulation. *Nat. Rev. Genet.* 5 522–531 10.1038/nrg1379 15211354

[B51] Hill J. J. Hashimoto T. Lewis D. A. (2006). Molecular mechanisms contributing to dendritic spine alterations in the prefrontal cortex of subjects with schizophrenia. *Mol. Psychiatry* 11 557–566 10.1038/sj.mp.4001792 16402129

[B52] Ide M. Lewis D. A. (2010). Altered cortical CDC42 signaling pathways in schizophrenia: implications for dendritic spine deficits. *Biol. Psychiatry* 68 25–32 10.1016/j.biopsych.2010.02.016 20385374 PMC2900524

[B53] Im H. I. Kenny P. J. (2012). MicroRNAs in neuronal function and dysfunction. *Trends Neurosci.* 35 325–334 10.1016/j.tins.2012.01.004 22436491 PMC3565236

[B54] Impey S. Davare M. Lesiak A. Fortin D. Ando H. Varlamova O. (2010). An activity-induced microRNA controls dendritic spine formation by regulating Rac1-PAK signaling. *Mol. Cell. Neurosci.* 43 146–156 10.1016/j.mcn.2009.10.005 19850129 PMC2818337

[B55] International Schizophrenia Consortium. (2008). Rare chromosomal deletions and duplications increase risk of schizophrenia. *Nature* 455 237–241 10.1038/nature07239 18668038 PMC3912847

[B56] Irie F. Yamaguchi Y. (2002). EphB receptors regulate dendritic spine development via intersectin, Cdc42 and N-WASP. *Nat. Neurosci.* 5 1117–1118 10.1038/nn964 12389031

[B57] Itoh M. Kim C. H. Palardy G. Oda T. Jiang Y. J. Maust D. (2003). Mind bomb is a ubiquitin ligase that is essential for efficient activation of Notch signaling by Delta. *Dev. Cell* 4 67–82 10.1016/S1534-5807(02)00409-4 12530964

[B58] Jiang K. Ren C. Nair V. D. (2013). MicroRNA-137 represses Klf4 and Tbx3 during differentiation of mouse embryonic stem cells. *Stem Cell Res.* 11 1299–1313 10.1016/j.scr.2013.09.001 24084696

[B59] John B. Enright A. J. Aravin A. Tuschl T. Sander C. Marks D. S. (2004). Human MicroRNA targets. *PLoS Biol.* 2:e363 10.1371/journal.pbio.0020363 PMC521178 15502875

[B60] Johnston R. J. Hobert O. (2003). A microRNA controlling left/right neuronal asymmetry in *Caenorhabditis elegans*. *Nature* 426 845–849 10.1038/nature02255 14685240

[B61] Karayiorgou M. Morris M. A. Morrow B. Shprintzen R. J. Goldberg R. Borrow J. (1995). Schizophrenia susceptibility associated with interstitial deletions of chromosome 22q11. *Proc. Natl. Acad. Sci. U.S.A.* 92 7612–7616 10.1073/pnas.92.17.7612 7644464 PMC41195

[B62] Karayiorgou M. Simon T. J. Gogos J. A. (2010). 22q11.2 microdeletions: linking DNA structural variation to brain dysfunction and schizophrenia. * Nat. Rev. Neurosci.* 11 402–416 10.1038/nrn2841 20485365 PMC2977984

[B63] Kim A. H. Parker E. K. Williamson V. Mcmichael G. O. Fanous A. H. Vladimirov V. I. (2012a). Experimental validation of candidate schizophrenia gene ZNF804A as target for hsa-miR-137. *Schizophr. Res.* 141 60–64 10.1016/j.schres.2012.06.038 22883350 PMC4104606

[B64] Kim S. Cho H. Lee D. Webster M. J. (2012b). Association between SNPs and gene expression in multiple regions of the human brain. *Transl. Psychiatry* 2 e113 10.1038/tp.2012.42 PMC3365261 22832957

[B65] Kim J. Inoue K. Ishii J. Vanti W. B. Voronov S. V. Murchison E. (2007). A MicroRNA feedback circuit in midbrain dopamine neurons. *Science* 317 1220–1224 10.1126/science.1140481 17761882 PMC2782470

[B66] Krichevsky A. M. Sonntag K. C. Isacson O. Kosik K. S. (2006). Specific microRNAs modulate embryonic stem cell-derived neurogenesis. *Stem Cells* 24 857–864 10.1634/stemcells.2005-0441 16357340 PMC2605651

[B67] Kurian S. M. Le-Niculescu H. Patel S. D. Bertram D. Davis J. Dike C. (2011). Identification of blood biomarkers for psychosis using convergent functional genomics. *Mol. Psychiatry* 16 37–58 10.1038/mp.2009.117 19935739

[B68] Kwon E. Wang W. Tsai L. H. (2013). Validation of schizophrenia-associated genes CSMD1, C10orf26, CACNA1C and TCF4 as miR-137 targets. *Mol. Psychiatry* 18 11–12 10.1038/mp.2011.170 22182936

[B69] Lee K. Kim J. H. Kwon O. B. An K. Ryu J. Cho K. (2012). An activity-regulated microRNA, miR-188, controls dendritic plasticity and synaptic transmission by downregulating neuropilin-2. *J. Neurosci.* 32 5678–5687 10.1523/JNEUROSCI.6471-11.2012 22514329 PMC5010781

[B70] Lett T. A. Chakravarty M. M. Felsky D. Brandl E. J. Tiwari A. K. Goncalves V. F. (2013). The genome-wide supported microRNA-137 variant predicts phenotypic heterogeneity within schizophrenia. *Mol. Psychiatry* 18 1146 10.1038/mp.2013.39 23459466

[B71] Leucht C. Stigloher C. Wizenmann A. Klafke R. Folchert A. Bally-Cuif L. (2008). MicroRNA-9 directs late organizer activity of the midbrain-hindbrain boundary. *Nat. Neurosci.* 11 641–648 10.1038/nn.2115 18454145

[B72] Levinson D. F. Duan J. Oh S. Wang K. Sanders A. R. Shi J. (2011). Copy number variants in schizophrenia: confirmation of five previous findings and new evidence for 3q29 microdeletions and VIPR2 duplications. *Am. J. Psychiatry* 168 302–316 10.1176/appi.ajp.2010.10060876 21285140 PMC4441324

[B73] Lippi G. Steinert J. R. Marczylo E. L. D’Oro S. Fiore R. Forsythe I. D. (2011). Targeting of the Arpc3 actin nucleation factor by miR-29a/b regulates dendritic spine morphology. *J. Cell Biol.* 194 889–904 10.1083/jcb.201103006 21930776 PMC3207289

[B74] Liu C. M. Wang R. Y. Saijilafu, Jiao Z. X. Zhang B. Y. Zhou F. Q. (2013). MicroRNA-138 and SIRT1 form a mutual negative feedback loop to regulate mammalian axon regeneration. *Genes Dev.* 27 1473–1483 10.1101/gad.209619.112 23796896 PMC3713428

[B75] Liu M. Lang N. Chen X. Tang Q. Liu S. Huang J. (2011). miR-185 targets RhoA and Cdc42 expression and inhibits the proliferation potential of human colorectal cells. *Cancer Lett.* 301 151–160 10.1016/j.canlet.2010.11.009 21186079

[B76] Lugli G. Torvik V. I. Larson J. Smalheiser N. R. (2008). Expression of microRNAs and their precursors in synaptic fractions of adult mouse forebrain. *J. Neurochem.* 106 650–661 10.1111/j.1471-4159.2008.05413.x 18410515 PMC3711666

[B77] Luikart B. W. Bensen A. L. Washburn E. K. Perederiy J. V. Su K. G. Li Y. (2011). miR-132 mediates the integration of newborn neurons into the adult dentate gyrus. *PLoS ONE* 6:e19077 10.1371/journal.pone.0019077 PMC3096628 21611182

[B78] Magill S. T. Cambronne X. A. Luikart B. W. Lioy D. T. Leighton B. H. Westbrook G. L. (2010). microRNA-132 regulates dendritic growth and arborization of newborn neurons in the adult hippocampus. *Proc. Natl. Acad. Sci. U.S.A.* 107 20382–20387 10.1073/pnas.1015691107 21059906 PMC2996687

[B79] Makeyev E. V. Zhang J. Carrasco M. A. Maniatis T. (2007). The microRNA miR-124 promotes neuronal differentiation by triggering brain-specific alternative pre-mRNA splicing. *Mol. Cell* 27 435–448 10.1016/j.molcel.2007.07.015 17679093 PMC3139456

[B80] Maller Schulman B. R. Liang X. Stahlhut C. Delconte C. Stefani G. Slack F. J. (2008). The let-7 microRNA target gene, Mlin41/Trim71 is required for mouse embryonic survival and neural tube closure. *Cell Cycle* 7 3935–3942 10.4161/cc.7.24.7397 19098426 PMC2895810

[B81] Mao Y. Mohan R. Zhang S. Tang X. (2013). MicroRNAs as pharmacological targets in diabetes. *Pharmacol. Res.* 75 37–47 10.1016/j.phrs.2013.06.005 23810798 PMC3786207

[B82] Mellios N. Sugihara H. Castro J. Banerjee A. Le C. Kumar A. (2011). miR-132, an experience-dependent microRNA, is essential for visual cortex plasticity. *Nat. Neurosci.* 14 1240–1242 10.1038/nn.2909 21892155 PMC3183341

[B83] Meola N. Gennarino V. A. Banfi S. (2009). microRNAs and genetic diseases. *Pathogenetics* 2 7 10.1186/1755-8417-2-7 PMC2778645 19889204

[B84] Moreau M. P. Bruse S. E. David-Rus R. Buyske S. Brzustowicz L. M. (2011). Altered microRNA expression profiles in postmortem brain samples from individuals with schizophrenia and bipolar disorder. *Biol. Psychiatry* 69 188–193 10.1016/j.biopsych.2010.09.039 21183010 PMC3038345

[B85] Muddashetty R. S. Nalavadi V. C. Gross C. Yao X. Xing L. Laur O. (2011). Reversible inhibition of PSD-95 mRNA translation by miR-125a, FMRP phosphorylation, and mGluR signaling. *Mol. Cell* 42 673–688 10.1016/j.molcel.2011.05.006 21658607 PMC3115785

[B86] Mukai J. Dhilla A. Drew L. J. Stark K. L. Cao L. Macdermott A. B. (2008). Palmitoylation-dependent neurodevelopmental deficits in a mouse model of 22q11 microdeletion. *Nat. Neurosci.* 11 1302–1310 10.1038/nn.2204 18836441 PMC2756760

[B87] Murphy K. C. Jones L. A. Owen M. J. (1999). High rates of schizophrenia in adults with velo-cardio-facial syndrome. *Arch. Gen. Psychiatry* 56 940–945 10.1001/archpsyc.56.10.940 10530637

[B88] Nakayama A. Y. Harms M. B. Luo L. (2000). Small GTPases Rac and Rho in the maintenance of dendritic spines and branches in hippocampal pyramidal neurons. *J. Neurosci.* 20 5329–5338 10884317 10.1523/JNEUROSCI.20-14-05329.2000 PMC6772334

[B89] Nishino J. Kim I. Chada K. Morrison S. J. (2008). Hmga2 promotes neural stem cell self-renewal in young but not old mice by reducing p16Ink4a and p19Arf expression. *Cell* 135 227–239 10.1016/j.cell.2008.09.017 18957199 PMC2582221

[B90] Nobes C. D. Hall A. (1995). Rho, rac, and cdc42 GTPases regulate the assembly of multimolecular focal complexes associated with actin stress fibers, lamellipodia, and filopodia. *Cell* 81 53–62 10.1016/0092-8674(95)90370-4 7536630

[B91] Nowakowski T. J. Fotaki V. Pollock A. Sun T. Pratt T. Price D. J. (2013). MicroRNA-92b regulates the development of intermediate cortical progenitors in embryonic mouse brain. *Proc. Natl. Acad. Sci. U.S.A.* 110 7056–7061 10.1073/pnas.1219385110 23569256 PMC3637761

[B92] O’Donovan M. C. Craddock N. Norton N. Williams H. Peirce T. Moskvina V. (2008). Identification of loci associated with schizophrenia by genome-wide association and follow-up. *Nat. Genet.* 40 1053–1055 10.1038/ng.201 18677311

[B93] Ossipova O. Ezan J. Sokol S. Y. (2009). PAR-1 phosphorylates Mind bomb to promote vertebrate neurogenesis. *Dev. Cell* 17 222–233 10.1016/j.devcel.2009.06.010 19686683 PMC2849776

[B94] Ouchi Y. Banno Y. Shimizu Y. Ando S. Hasegawa H. Adachi K. (2013). Reduced adult hippocampal neurogenesis and working memory deficits in the Dgcr8-deficient mouse model of 22q11.2 deletion-associated schizophrenia can be rescued by IGF2. *J. Neurosci.* 33 9408–9419 10.1523/JNEUROSCI.2700-12.2013 23719809 PMC6618567

[B95] Packer A. N. Xing Y. Harper S. Q. Jones L. Davidson B. L. (2008). The bifunctional microRNA miR-9/miR-9* regulates REST and CoREST and is downregulated in Huntington’s disease. *J. Neurosci.* 28 14341–14346 10.1523/JNEUROSCI.2390-08.2008 19118166 PMC3124002

[B96] Pathania M. Torres-Reveron J. Yan L. Kimura T. Lin T. V. Gordon V. (2012). miR-132 enhances dendritic morphogenesis, spine density, synaptic integration, and survival of newborn olfactory bulb neurons. *PLoS ONE* 7:e38174 10.1371/journal.pone.0038174 PMC3364964 22693596

[B97] Peng C. Li N. Ng Y. K. Zhang J. Meier F. Theis F. J. (2012). A unilateral negative feedback loop between miR-200 microRNAs and Sox2/E2F3 controls neural progenitor cell-cycle exit and differentiation. *J. Neurosci.* 32 13292–13308 10.1523/JNEUROSCI.2124-12.2012 22993445 PMC3752087

[B98] Philip N. Bassett A. (2011). Cognitive, behavioural and psychiatric phenotype in 22q11.2 deletion syndrome. * Behav. Genet.* 41 403–412 10.1007/s10519-011-9468-z 21573985 PMC3139630

[B99] Powell S. B. Zhou X. Geyer M. A. (2009). Prepulse inhibition and genetic mouse models of schizophrenia. *Behav. Brain Res.* 204 282–294 10.1016/j.bbr.2009.04.021 19397931 PMC2735602

[B100] Reddy-Alla S. Schmitt B. Birkenfeld J. Eulenburg V. Dutertre S. Bohringer C. (2010). PH-domain-driven targeting of collybistin but not Cdc42 activation is required for synaptic gephyrin clustering. *Eur. J. Neurosci.* 31 1173–1184 10.1111/j.1460-9568.2010.07149.x 20345913

[B101] Reid T. Bathoorn A. Ahmadian M. R. Collard J. G. (1999). Identification and characterization of hPEM-2, a guanine nucleotide exchange factor specific for Cdc42. *J. Biol. Chem.* 274 33587–33593 10.1074/jbc.274.47.33587 10559246

[B102] Remenyi J. Van Den Bosch M. W. Palygin O. Mistry R. B. Mckenzie C. Macdonald A. (2013). miR-132/212 knockout mice reveal roles for these miRNAs in regulating cortical synaptic transmission and plasticity. *PLoS ONE* 8:e62509 10.1371/journal.pone.0062509 PMC3637221 23658634

[B103] Rinaldi A. Vincenti S. De Vito F. Bozzoni I. Oliverio A. Presutti C. (2010). Stress induces region specific alterations in microRNAs expression in mice. *Behav. Brain Res.* 208 265–269 10.1016/j.bbr.2009.11.012 19913057

[B104] Robin N. H. Shprintzen R. J. (2005). Defining the clinical spectrum of deletion 22q11.2. *J. Pediatr.* 147 90–96 10.1016/j.jpeds.2005.03.007 16027702

[B105] Rybak A. Fuchs H. Smirnova L. Brandt C. Pohl E. E. Nitsch R. (2008). A feedback loop comprising lin-28 and let-7 controls pre-let-7 maturation during neural stem-cell commitment. *Nat. Cell Biol.* 10 987–993 10.1038/ncb1759 18604195

[B106] Saba R. Storchel P. H. Aksoy-Aksel A. Kepura F. Lippi G. Plant T. D. (2012). Dopamine-regulated microRNA MiR-181a controls GluA2 surface expression in hippocampal neurons. *Mol. Cell. Biol.* 32 619–632 10.1128/MCB.05896-11 22144581 PMC3266602

[B107] Sanuki R. Onishi A. Koike C. Muramatsu R. Watanabe S. Muranishi Y. (2011). miR-124a is required for hippocampal axogenesis and retinal cone survival through Lhx2 suppression. *Nat. Neurosci.* 14 1125–1134 10.1038/nn.2897 21857657

[B108] Sathyan P. Golden H. B. Miranda R. C. (2007). Competing interactions between micro-RNAs determine neural progenitor survival and proliferation after ethanol exposure: evidence from an ex vivo model of the fetal cerebral cortical neuroepithelium. *J. Neurosci.* 27 8546–8557 10.1523/JNEUROSCI.1269-07.2007 17687032 PMC2915840

[B109] Sawa A. Snyder S. H. (2002). Schizophrenia: diverse approaches to a complex disease. *Science* 296 692–695 10.1126/science.1070532 11976442

[B110] Schizophrenia Psychiatric Genome-Wide Association Study (GWAS) Consortium (2011). Genome-wide association study identifies five new schizophrenia loci. *Nat. Genet.* 43 969–976 10.1038/ng.940 21926974 PMC3303194

[B111] Schofield C. M. Hsu R. Barker A. J. Gertz C. C. Blelloch R. Ullian E. M. (2011). Monoallelic deletion of the microRNA biogenesis gene Dgcr8 produces deficits in the development of excitatory synaptic transmission in the prefrontal cortex. *Neural Dev.* 6 11 10.1186/1749-8104-6-11 PMC3082233 21466685

[B112] Schratt G. (2009). microRNAs at the synapse. *Nat. Rev. Neurosci.* 10 842–849 10.1038/nrn2763 19888283

[B113] Schratt G. M. Tuebing F. Nigh E. A. Kane C. G. Sabatini M. E. Kiebler M. (2006). A brain-specific microRNA regulates dendritic spine development. *Nature* 439 283–289 10.1038/nature04367 16421561

[B114] Schreiner M. J. Lazaro M. T. Jalbrzikowski M. Bearden C. E. (2013). Converging levels of analysis on a genomic hotspot for psychosis: insights from 22q11.2 deletion syndrome. * Neuropharmacology* 68 157–173 10.1016/j.neuropharm.2012.09.012 23098994 PMC3677073

[B115] Scott H. L. Tamagnini F. Narduzzo K. E. Howarth J. L. Lee Y. B. Wong L. F. (2012). MicroRNA-132 regulates recognition memory and synaptic plasticity in the perirhinal cortex. *Eur. J. Neurosci.* 36 2941–2948 10.1111/j.1460-9568.2012.08220.x 22845676 PMC3488600

[B116] Shaikh T. H. Kurahashi H. Saitta S. C. O’Hare A. M Hu P. Roe B. A. (2000). Chromosome 22-specific low copy repeats and the 22q11.2 deletion syndrome: genomic organization and deletion endpoint analysis. * Hum. Mol. Genet.* 9 489–501. 10.1093/hmg/9.4.489 10699172

[B117] Shibata M. Kurokawa D. Nakao H. Ohmura T. Aizawa S. (2008). MicroRNA-9 modulates Cajal–Retzius cell differentiation by suppressing Foxg1 expression in mouse medial pallium. *J. Neurosci.* 28 10415–10421 10.1523/JNEUROSCI.3219-08.2008 18842901 PMC6671033

[B118] Shibata M. Nakao H. Kiyonari H. Abe T. Aizawa S. (2011). MicroRNA-9 regulates neurogenesis in mouse telencephalon by targeting multiple transcription factors. *J. Neurosci.* 31 3407–3422 10.1523/JNEUROSCI.5085-10.2011 21368052 PMC6623912

[B119] Siegel G. Obernosterer G. Fiore R. Oehmen M. Bicker S. Christensen M. (2009). A functional screen implicates microRNA-138-dependent regulation of the depalmitoylation enzyme APT1 in dendritic spine morphogenesis. *Nat. Cell Biol.* 11 705–716 10.1038/ncb1876 19465924 PMC3595613

[B120] Smrt R. D. Szulwach K. E. Pfeiffer R. L. Li X. Guo W. Pathania M. (2010). MicroRNA miR-137 regulates neuronal maturation by targeting ubiquitin ligase mind bomb-1. *Stem Cells* 28 1060–1070 10.1002/stem.431 20506192 PMC3140955

[B121] Soriano A. Jubierre L. Almazan-Moga A. Molist C. Roma J. De Toledo J. S. (2013). microRNAs as pharmacological targets in cancer. *Pharmacol. Res.* 75 3–14 10.1016/j.phrs.2013.03.006 23537752

[B122] Sosanya N. M. Huang P. P. Cacheaux L. P. Chen C. J. Nguyen K. Perrone-Bizzozero N. I. (2013). Degradation of high affinity HuD targets releases Kv1.1 mRNA from miR-129 repression by mTORC1. *J. Cell Biol.* 202 53–69 10.1083/jcb.201212089 23836929 PMC3704988

[B123] Stark K. L. Xu B. Bagchi A. Lai W. S. Liu H. Hsu R. (2008). Altered brain microRNA biogenesis contributes to phenotypic deficits in a 22q11-deletion mouse model. *Nat. Genet.* 40 751–760 10.1038/ng.138 18469815

[B124] Stefansson H. Rujescu D. Cichon S. Pietilainen O. P. Ingason A. Steinberg S. (2008). Large recurrent microdeletions associated with schizophrenia. *Nature* 455 232–236 10.1038/nature07229 18668039 PMC2687075

[B125] Sullivan P. F. Daly M. J O’Donovan M. (2012). Genetic architectures of psychiatric disorders: the emerging picture and its implications. *Nat. Rev. Genet.* 13 537–551 10.1038/nrg3240 22777127 PMC4110909

[B126] Tada T. Sheng M. (2006). Molecular mechanisms of dendritic spine morphogenesis. *Curr. Opin. Neurobiol.* 16 95–101 10.1016/j.conb.2005.12.001 16361095

[B127] Tamminga C. A. Stan A. D. Wagner A. D. (2010). The hippocampal formation in schizophrenia. *Am. J. Psychiatry* 167 1178–1193 10.1176/appi.ajp.2010.09081187 20810471

[B128] Tognini P. Putignano E. Coatti A. Pizzorusso T. (2011). Experience-dependent expression of miR-132 regulates ocular dominance plasticity. *Nat. Neurosci.* 14 1237–1239 10.1038/nn.2920 21892154 PMC3183093

[B129] Tomari Y. Zamore P. D. (2005). MicroRNA biogenesis: drosha can’t cut it without a partner. *Curr. Biol.* 15 R61–R64 10.1016/j.cub.2004.12.057 15668159

[B130] Ursu S. Kring A. M. Gard M. G. Minzenberg M. J. Yoon J. H. Ragland J. D. (2011). Prefrontal cortical deficits and impaired cognition–emotion interactions in schizophrenia. *Am. J. Psychiatry* 168 276–285 10.1176/appi.ajp.2010.09081215 21205806 PMC4019338

[B131] Visvanathan J. Lee S. Lee B. Lee J. W. Lee S. K. (2007). The microRNA miR-124 antagonizes the anti-neural REST/SCP1 pathway during embryonic CNS development. *Genes Dev.* 21 744–749 10.1101/gad.1519107 17403776 PMC1838526

[B132] Vo N. Klein M. E. Varlamova O. Keller D. M. Yamamoto T. Goodman R. H. (2005). A cAMP-response element binding protein-induced microRNA regulates neuronal morphogenesis. *Proc. Natl. Acad. Sci. U.S.A.* 102 16426–16431 10.1073/pnas.0508448102 16260724 PMC1283476

[B133] Wang R. Y. Phang R. Z. Hsu P. H. Wang W. H. Huang H. T. Liu I. Y. (2013). In vivo knockdown of hippocampal miR-132 expression impairs memory acquisition of trace fear conditioning. *Hippocampus* 23 625–633 10.1002/hipo.22123 23520022

[B134] Wayman G. A. Davare M. Ando H. Fortin D. Varlamova O. Cheng H. Y. (2008). An activity-regulated microRNA controls dendritic plasticity by down-regulating p250GAP. *Proc. Natl. Acad. Sci. U.S.A.* 105 9093–9098 10.1073/pnas.0803072105 18577589 PMC2449370

[B135] Wegner A. M. Nebhan C. A. Hu L. Majumdar D. Meier K. M. Weaver A. M. (2008). N-wasp and the arp2/3 complex are critical regulators of actin in the development of dendritic spines and synapses. *J. Biol. Chem.* 283 15912–15920 10.1074/jbc.M801555200 18430734 PMC2414292

[B136] Williams H. J. Norton N. Dwyer S. Moskvina V. Nikolov I. Carroll L. (2011). Fine mapping of ZNF804A and genome-wide significant evidence for its involvement in schizophrenia and bipolar disorder. *Mol. Psychiatry* 16 429–441 10.1038/mp.2010.36 20368704 PMC3918934

[B137] Xu B. Hsu P. K. Stark K. L. Karayiorgou M. Gogos J. A. (2013). Derepression of a neuronal inhibitor due to miRNA dysregulation in a schizophrenia-related microdeletion. *Cell* 152 262–275 10.1016/j.cell.2012.11.052 23332760 PMC3556818

[B138] Xu B. Karayiorgou M. Gogos J. A. (2010). MicroRNAs in psychiatric and neurodevelopmental disorders. *Brain Res.* 1338 78–88 10.1016/j.brainres.2010.03.109 20388499 PMC2883644

[B139] Yagi H. Furutani Y. Hamada H. Sasaki T. Asakawa S. Minoshima S. (2003). Role of TBX1 in human del22q11.2 syndrome. *Lancet* 362 1366–1373 10.1016/S0140-6736(03)14632-6 14585638

[B140] Yang D. Li T. Wang Y. Tang Y. Cui H. Zhang X. (2012). miR-132 regulates the differentiation of dopamine neurons by directly targeting Nurr1 expression. *J. Cell Sci.* 125 1673–1682 10.1242/jcs.086421 22328530

[B141] Yoo A. S. Staahl B. T. Chen L. Crabtree G. R. (2009). MicroRNA-mediated switching of chromatin-remodelling complexes in neural development. *Nature* 460 642–646 10.1038/nature08139 19561591 PMC2921580

[B142] Yoo A. S. Sun A. X. Li L. Shcheglovitov A. Portmann T. Li Y. (2011). MicroRNA-mediated conversion of human fibroblasts to neurons. *Nature* 476 228–231 10.1038/nature10323 21753754 PMC3348862

[B143] Yu J. Y. Chung K. H. Deo M. Thompson R. C. Turner D. L. (2008). MicroRNA miR-124 regulates neurite outgrowth during neuronal differentiation. *Exp. Cell Res.* 314 2618–2633 10.1016/j.yexcr.2008.06.002 18619591 PMC2702206

[B144] Zeller T. Wild P. Szymczak S. Rotival M. Schillert A. Castagne R. (2010). Genetics and beyond – the transcriptome of human monocytes and disease susceptibility. *PLoS ONE* 5:e10693 10.1371/journal.pone.0010693 PMC2872668 20502693

[B145] Zhang J. Liu L. H. Zhou Y. Li Y. P. Shao Z. H. Wu Y. J. (2011). Effects of miR-541 on neurite outgrowth during neuronal differentiation. *Cell Biochem. Funct.* 29 279–286 10.1002/cbf.1747 21452340

[B146] Zhang Y. Ueno Y. Liu X. S. Buller B. Wang X. Chopp M. (2013). The MicroRNA-17-92 cluster enhances axonal outgrowth in embryonic cortical neurons. *J. Neurosci.* 33 6885–6894 10.1523/JNEUROSCI.5180-12.2013 23595747 PMC3657758

[B147] Zhao C. Sun G. Li S. Lang M. F. Yang S. Li W. (2010). MicroRNA let-7b regulates neural stem cell proliferation and differentiation by targeting nuclear receptor TLX signaling. *Proc. Natl. Acad. Sci. U.S.A.* 107 1876–1881 10.1073/pnas.0908750107 20133835 PMC2836616

[B148] Zhao C. Sun G. Li S. Shi Y. (2009). A feedback regulatory loop involving microRNA-9 and nuclear receptor TLX in neural stem cell fate determination. *Nat. Struct. Mol. Biol.* 16 365–371 10.1038/nsmb.1576 19330006 PMC2667220

[B149] Zovoilis A. Agbemenyah H. Y. Agis-Balboa R. C. Stilling R. M. Edbauer D. Rao P. (2011). microRNA-34c is a novel target to treat dementias. *EMBO J.* 30 4299–4308 10.1038/emboj.2011.327 21946562 PMC3199394

[B150] Zweier C. Sticht H. Aydin-Yaylagul I. Campbell C. E. Rauch A. (2007). Human TBX1 missense mutations cause gain of function resulting in the same phenotype as 22q11.2 deletions. *Am. J. Hum. Genet.* 80 510–517 10.1086/511993 17273972 PMC1821102

